# Hsa_circRNA_0017620 regulated cell progression of non‐small‐cell lung cancer via miR‐520a‐5p/KRT5 axis

**DOI:** 10.1002/jcla.24347

**Published:** 2022-03-18

**Authors:** Shan Chen, Kelin Hong, Long Zhou, Ruizhi Ran, Jinqi Huang, Yong Zheng, Maohui Xing, Yanli Cai

**Affiliations:** ^1^ Department of Oncology The Central Hospital of Enshi Tujia and Miao Autonomous Prefecture Enshi China; ^2^ Department of Cardiothoracic Surgery The Central Hospital of Enshi Tujia and Miao Autonomous Prefecture Enshi China; ^3^ Department of Pulmonary and Critical Care Medicine The Central Hospital of Enshi Tujia and Miao Autonomous Prefecture Enshi China

**Keywords:** hsa_circ_0017620, KRT5, miR‐520a‐5p, NSCLC, tumorigenicity

## Abstract

**Background:**

CircRNA is a very important functional RNA that plays an important role in the development and metabolism of cancer. However, the study of circRNA in NSCLC has not been fully elucidated.

**Methods:**

The expression of hsa_circ_0017620, SFMBT2, miR‐520a‐5p, and KRT5 was determined using qRT‐PCR. KRT5, Twist1, E‐cadherin, and Ki67 protein expression were measured with western blot. The positive expression rates of Ki67 and Vimentin were determined by immunohistochemistry assay. 5‐Ethynyl‐2’‐deoxyuridine (EdU), colony formation, and MTT assays were used to assess cell proliferation. Transwell migration and invasion assay were applied to determine cell migration and invasion. Dual‐luciferase reporter and RNA immunoprecipitation assays were used to verify the relationship among hsa_circ_0017620, miR‐520a‐5p, and KRT5. The animal experiment was used to ensure the effects of hsa_circ_0017620 on tumor growth *in vivo*.

**Results:**

Hsa_circ_0017620 was upregulated in NSCLC cells and tissues. MiR‐520a‐5p had been verified to be a target miRNA of hsa_circ_0017620 and KRT5 had been verified to be a target mRNA of miR‐520a‐5p in NSCLC cells. Knockdown of hsa_circ_0017620 inhibited cell proliferation, migration, and invasion in NSCLC cells, which was reversed by downregulating miR‐520a‐5p or upregulating KRT5 in NSCLC. Overexpression of hsa_circ_0017620 had opposite effects in NSCLC. Moreover, hsa_circ_0017620 silencing inhibited tumor growth *in vivo* of NSCLC.

**Conclusion:**

In this study, we found that hsa_circ_0017620 played an important role in NSCLC progression. Hsa_circ_0017620 regulated cell proliferation, invasion, and migration through targeting miR‐520a‐5p/KRT5 axis in NSCLC, providing a potential new target for the treatment and diagnosis of NSCLC.

## Graphical abstract

Hsa_circ_0017620 regulated cell proliferation, migration, and invasion through targeting miR‐520a‐5p in NSCLC. (A and B) The expression of miR‐520a‐5p was detected in sh‐NC, sh‐hsa_circ_0017620, sh‐hsa_circ_0017620+anti‐miR‐NC, and sh‐hsa_circ_0017620+anti‐miR‐520a‐5p groups with qRT‐PCR in A549 and H460 cells. (C) DNA synthesis was analyzed by EdU assay in A549 and H460 cells transfected with sh‐NC, sh‐hsa_circ_0017620, sh‐hsa_circ_0017620+anti‐miR‐NC, and sh‐hsa_circ_0017620+anti‐miR‐520a‐5p. (D) The number of colony cells was measured in sh‐NC, sh‐hsa_circ_0017620, sh‐hsa_circ_0017620+anti‐miR‐NC, and sh‐hsa_circ_0017620+anti‐miR‐520a‐5p groups using colony formation assay in A549 and H460 cells. (E and F) Cell proliferation was measured using MTT assay in sh‐NC, sh‐hsa_circ_0017620, sh‐hsa_circ_0017620+anti‐miR‐NC, and sh‐hsa_circ_0017620+anti‐miR‐520a‐5p groups in A549 and H460 cells. (G) The protein expression of Ki67 was detected with western blot in sh‐NC, sh‐hsa_circ_0017620, sh‐hsa_circ_0017620+anti‐miR‐NC, and sh‐hsa_circ_0017620+anti‐miR‐520a‐5p groups in A549 and H460 cells. (H and I) Cell migration and invasion were measured with transwell assay in sh‐NC, sh‐hsa_circ_0017620, sh‐hsa_circ_0017620+anti‐miR‐NC, and sh‐hsa_circ_0017620+anti‐miR‐520a‐5p groups in A549 and H460 cells. (J and K) The protein expression of Twist1 and E‐cadherin was detected with western blot in sh‐NC, sh‐hsa_circ_0017620, sh‐hsa_circ_0017620+anti‐miR‐NC, and sh‐hsa_circ_0017620+anti‐miR‐520a‐5p groups in A549 and H460 cells. *p<0.05.

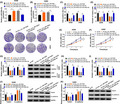

## 1 INTRODUCTION

Lung cancer is malignant tumor that threats to human life and health, which has the fastest‐growing morbidity and mortality rates.^1^, ^2^ In spite of tremendous efforts in improving therapy, the survival rate of patients with the disease is dissatisfactory. However, the etiology and mechanism of lung cancer remain unknown. In fact, NSCLC accounts for about 85% of all lung cancers^3^; however, research on the pathogenesis and treatment of NSCLC is still not completely clear.

Circular RNAs (circRNAs) are endogenous transcripts, and play important roles in cancer progression.^4^, ^5^, ^6^, ^7^ Moreover, circRNA is a closed‐loop structure, which is less susceptible to the effects of exonucleases and is more functionally stable.^8^, ^9^ For example, hsa_circRNA_100876 was overexpressed in NSCLC and was associated with prognostic.^10^ Otherwise, hsa_circ_0001649 acted as a novel biomarker, and was down expressed in hepatocellular carcinoma tissues and was correlated with tumor size.^11^ In gastric cancer (GC), hsa_circ_0000467 enhanced cancer progression and was related to diagnostic and prognostic.^12^ Besides, hsa_circ_0017620 was overexpressed in GC tissues and promote cell proliferation.^13^ Not only that, but circRNAs can also bind miRNAs to regulate mRNA affect cell metabolism and development in various cancers.^14^ For example, hsa_circRNA_103595 promoted lung cancer cell proliferation and invasion.^15^ However, the underlying regulatory mechanism of hsa_circ_0017620 in NSCLC has not been fully clarified.

In the work, hsa_circ_0017620 was overexpressed in NSCLC tissues, and acted as a competing endogenous RNA to bind to miR‐520a‐5p to regulate KRT5 expression, so as to mediate NSCLC cell malignancy.

## 2 METHODS

### 2.1 Patients and samples

Eighty‐eight pairs of NSCLC tissues and adjacent normal tissues were collected from patients who were diagnosed with NSCLC in the Central Hospital of Enshi Tujia and Miao Autonomous Prefecture. This study was approved by the ethics committee of the Central Hospital of Enshi Tujia and Miao Autonomous Prefecture. Written informed consent was obtained from all patients and their guardians. All samples were collected and stored at −80℃ for the following experiments.

### 2.2 Cell culture and transfection

Normal lung cell lines (HBE) and NSCLC cell lines (A549, HCC827, H460, and Calu‐3) were purchased from the Meixuan Biotech (Shanghai, China) or Wanwu Biotech (Hefei, China), and cultured in DMEM (Gibco‐BRL, Rockville, IN, USA) with 10% fetal bovine serum (FBS) and 1% penicillin/streptomycin at 37℃ with 5% CO_2_.

Scrambled shRNA of hsa_circ_0017620 (sh‐hsa_circ_0017620) or its negative control (sh‐NC), and pcDNA3.1‐hsa_circ_0017620/KRT5 (hsa_circ_0017620/KRT5) or its negative control (Vector/pcDNA) were obtained from GenePharma Technology (Shanghai, China). miR‐520a‐5p inhibitor (anti‐miR‐520a‐5p), miR‐520a‐5p mimic (miR‐520a‐5p), and their negative control (anti‐miR‐NC and miR‐NC) were purchased from GenePharma Technology. All plasmid and oligos were transfected into A549 and H460 cells using Polyplus‐transfection^®^ (Illkirch, France). In an animal experiment, full‐length hsa_circ_0017620 were subcloned into LV5 lentiviruses and infected into A549 cells, named sh‐hsa_circ_0017620. The Sh‐NC or sh‐hsa_circ_0017620 were purchased from GenePharma Technology.

### 2.3 qRT‐PCR and RNase R treatment

Total RNA was extracted using TRIzol reagent (Yeasen Biotech, Shanghai, China). NanoDrop 2000c was used to measure RNA quality. RNase R treatment was performed for 20 min at 37°C using 3 U/mg RNase R (Epicenter Biotechnologies, Madison, Wisconsin, USA). For hsa_circ_0017620, SFMBT2 expression, and KRT5, 500 ng of RNA was reversed transcribed into cDNA using cDNA Synthesis reagents with gDNA digester plus (Yeasen Biotech). For miR‐520a‐5p expression, cDNA synthesis kit (Thermo Fisher, Waltham, MA, USA) was used to detect. Quantitative RT‐PCR was performed with the use of SYBR Green Master Mix (Yeasen Biotech). Primer sequences are shown in Table 1.

**TABLE 1:** Primer sequences used for qRT‐PCR

Name		Primer sequences (5′−3′)
Hsa_circ_0017620	Forward	TCTCCTGCGTCGGTGACTAA
	Reverse	CTCGCAGAGGACCTTCCAGG
SFMBT2	Forward	GAACAACCCGGACACGTACT
	Reverse	GCTGTCCTCGAACCAGTCAA
miR‐520a‐5p	Forward	GTATGACTCCAGAGGGAAG
	Reverse	CTCAACTGGTGTCGTGGAGT
KRT5	Forward	CAGTGGAGAAGGAGTTGGACC
	Reverse	TGCTGCTGGAGTAGTAGCTT
GAPDH	Forward	GGTCACCAGGGCTGCTTT
	Reverse	GGAAGATGGTGATGGGATT
U6	Forward	TCGCTTCGGCAGCACATA
	Reverse	TTTGCGTGTCATCCTTGC

### 2.4 Analysis for cell proliferation

For EdU assay, transfected NSCLC cells were cultured for 48 h and passaged in 96‐well plates, which were supplied with 50 μM EdU‐labeled medium. Then, cDNA synthesis detection kit (Ribobio, Guangzhou, China) was conducted to analyze cell proliferation. Fluorescence microscope (Olympus, Tokyo, Japan) was used to analyze samples.

For colony formation assay, as shown previously,^16^ NSCLC cells in six‐well plates went through transfection. After 2‐week culture, colonies were fixed with 10% formaldehyde (Aladdin, Shanghai, China), and then incubated with 0.5% crystal violet (XYbscience, Shanghai, China). A light microscope was used to count the number of the colonies.

For MTT assay, transfected cells were seeded into 35‐mm Petri dishes and went through 24‐hour culture. Then, cells were exposed to 20‐μL MTT assay and incubated for 3 h. After incubating with DMSO, samples were detected at 490 nm using an enzyme‐linked immune detector.

### 2.5 Western blot

The proteins were isolated through using SDS‐PAGE, and then the separated proteins were transferred onto PVDF membrane. The membrane was incubated with primary antibodies, anti‐KRT5 (1:2000, Proteintech, Chicago, USA), anti‐GAPDH (1:1000, Proteintech), anti‐Ki67 (1:1500, Proteintech), anti‐Twist1 (1:2000, Proteintech), and anti‐E‐cadherin (1:2000, Proteintech) at 4 °C overnight. The blots were exposed using enhanced chemiluminescence (ECL) system (Thermo Fisher).

### 2.6 Transwell migration and invasion assay

Transfected cells were seeded onto the upper chamber with Matrigel for invasion assay, transfected cells were seeded onto the upper chamber without Matrigel for migration assay. The lower chamber was added into 500 μL medium. After incubation for 2 h, transfected cells in lower chamber were fixed with 75% methanol and stained with crystal violet. Finally, cells were counted under a microscope.

### 2.7 Dual‐luciferase reporter assay

The wild‐type and mutant fragments of hsa_circ_0017620 or KRT5 containing putative binding sites of miR‐520a‐5p were amplified and inserted into the psiCHECK‐2 vector. Then, the hsa_circ_0017620‐wt or hsa_circ_0017620‐mut was cotransfected with miR‐NC or miR‐520a‐5p into A549 and H460 cells using Polyplus‐transfection^®^. Similarly, the KRT5‐wt or KRT5‐mut was cotransfected with miR‐NC or miR‐520a‐5p into A549 and H460 cells using Polyplus‐transfection^®^. Then, luciferase activities were detected using Dual‐Lucy Assay Kit (Keygen, Nanjing, China).

### 2.8 RNA immunoprecipitation assay

According to the guidebook of Magna RIP kit (Millipore, Bedford, MA, USA), A549 and H460 cells were lysed using RNA immunoprecipitation (RIP) buffer, and the lysates were incubated with magnetic beads, which were pre‐incubated with Ago2 antibody and IgG antibody for 30 min. After centrifugation at 12000 g, immunoprecipitated RNA was analyzed by qRT‐PCR.

### 2.9 Animal experiment

The animal experiment was approved by the Ethical Committee for Animal Research of the Central Hospital of Enshi Tujia and Miao Autonomous Prefecture. Six BALB/c female nude mice (4–5 weeks) were purchased from Animal Center of Central South University and randomly divided into two groups. Sh‐NC or sh‐hsa_circ_0017620 (2 × 10^6^/0.2 ml PBS) were injected subcutaneously into the flank of mice. Tumor volume was measured every 5 days after transfection of 10 days. After transfection for 30 days, the mice were then killed and tumor weight was measured.

## 3 IMMUNOHISTOCHEMISTRY ANALYSIS

Immunohistochemistry (IHC) assay was carried out referring to the manufacturer's instruction. Briefly, paraffin‐embedded tumors were fixed and dehydrated. Next, antigen retrieval for the sections immersed in sodium citrate was carried out at 80°C. The sections were incubated with Ki67 and Vimentin antibodies (1:100; Affinity, Nanjing, China), and 3,3‐diaminobenzidine (DAB) substrate, followed by counterstaining with hematoxylin. CX31‐LV320 microscope (Olympus) was employed to capture images.

### 3.1 Statistical analysis

All data were presented as mean ± standard deviation. Data analysis was performed using GraphPad Prism software. The statistical significance of the two groups was analyzed using the Student's *t*‐test with two tails. The statistical significance of three or more groups was analyzed using one‐way ANOVA. *p*‐value <0.05 was considered significant.

## 4 RESULTS

### 4.1 Hsa_circ_0017620 expression was upregulated in NSCLC tissue and cells

**FIGURE 1:**
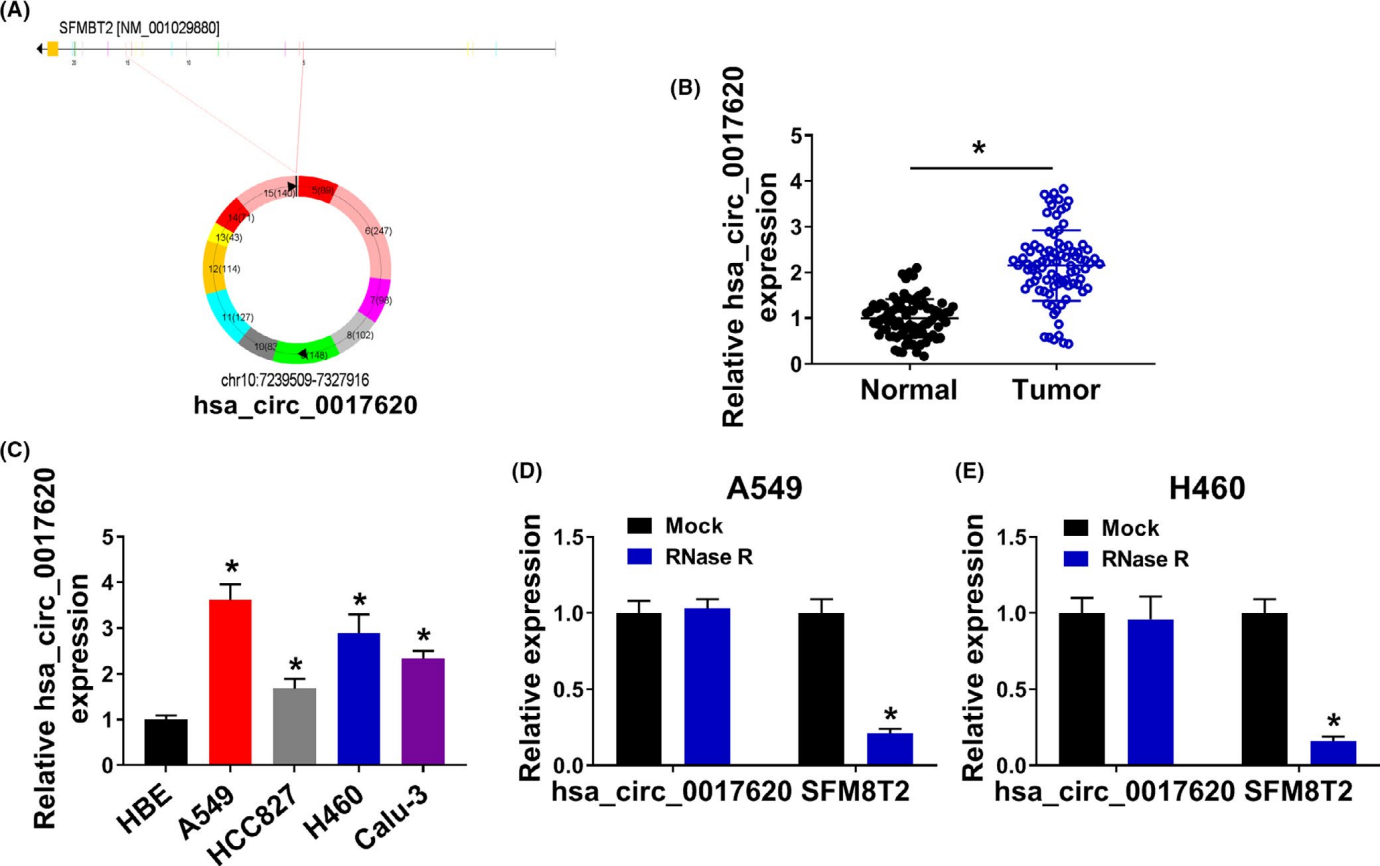
Hsa_circ_0017620 expression was upregulated in NSCLC tissue and cells. (A) The generation of hsa_circ_0017620. (B) The expression of hsa_circ_0017620 was detected in normal tissues and NSCLC tissues with qRT‐PCR. (C) The expression of hsa_circ_0017620 was detected in normal cells (HBE) and NSCLC cells (A549, HCC827, H460, and Calu‐3) with qRT‐PCR. (D and E) qRT‐PCR analysis of hsa_circ_0017620 and SFMBT2 mRNA expression after treatment with RNase R in A549 and H460 cells. *p < 0.05

Hsa_circ_0017620 was generated by cyclization of exons 5–15 of SFMBT2 gene (Figure 1A). We performed qRT‐PCR on the collected NSCLC tissues and adjacent tissues (N=54), and found that the expression of hsa_circ_0017620 in tumor tissues was significantly higher than that in adjacent tissues (Figure 1B). Also, we detected the expression of hsa_circ_0017620 in normal lung cells (HEB) and NSCLC cells (A549, HCC827, H460, and Calu‐3) using qRT‐PCR. The results showed that compared with HEB cells, hsa_circ_0017620 expression was significantly increased in A549, HCC827, H460, and Calu‐3 cells (Figure 1C). We selected two NSCLC cell lines (A549 and H460) with higher hsa_circ_0017620 expression for subsequent experiments. After RNA was processed by RNase R, the expression of hsa_circ_0017620 was not sensitive to RNase R, while the expression of linear SFMBT2 was significantly reduced in A549 and H460 cells (Figure 1D,E). Therefore, hsa_circ_0017620 was highly expressed in NSCLC and might play a role in the cell progression of NSCLC.

### 4.2 Hsa_circ_0017620 regulated cell proliferation of NSCLC

**FIGURE 2:**
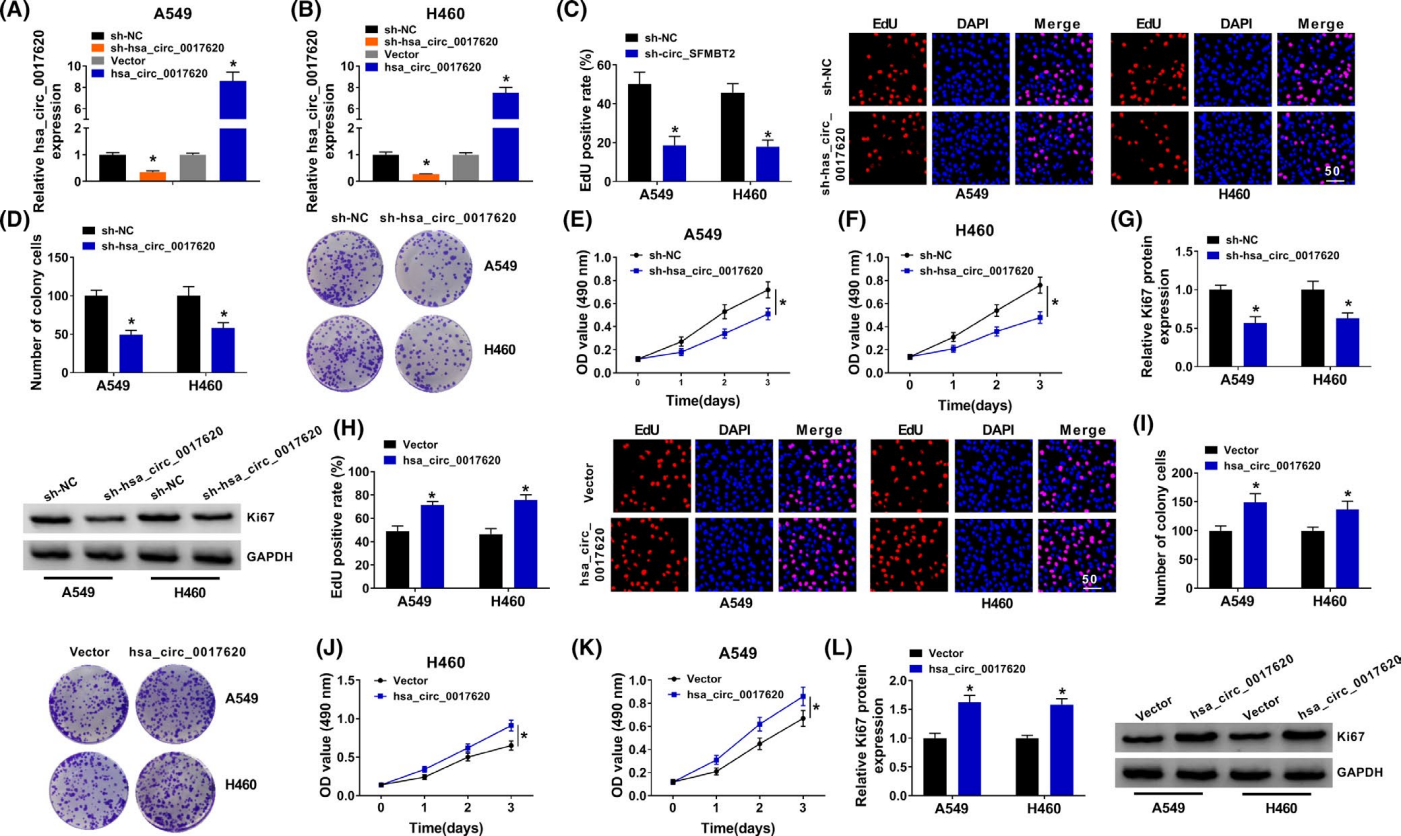
Hsa_circ_0017620 regulated cell proliferation of NSCLC. (A and B) The expression of hsa_circ_0017620 was detected in si‐NC, si‐hsa_circ_0017620, Vector, and hsa_circ_0017620 groups with qRT‐PCR in A549 and H460 cells. (C and H) DNA synthesis in A549 and H460 cells was detected by EdU assay in A549 and H460 cells transfected with si‐NC, si‐hsa_circ_0017620, Vector, and hsa_circ_0017620. (D and I) The number of colony cells was measured in si‐NC and si‐hsa_circ_0017620, vector, and hsa_circ_0017620 groups with colony formation assay in A549 and H460 cells. (E, F, J, K) Cell proliferation was measured in si‐NC, si‐hsa_circ_0017620, Vector, and hsa_circ_0017620 groups with MTT assay in A549 and H460 cells. (G and L) The expression of Ki67 was detected in si‐NC, si‐hsa_circ_0017620, Vector, and hsa_circ_0017620 groups with western blot in A549 and H460 cells. *p < 0.05

To ensure the function of hsa_circ_0017620 in NSCLC, we obtained the A549 and H460 cells transfected with si‐NC, si‐hsa_circ_0017620, Vector or hsa_circ_0017620, and qRT‐PCR determined that si‐hsa_circ_0017620 could inhibit hsa_circ_0017620 expression, while hsa_circ_0017620 could promote hsa_circ_0017620 expression (Figure 2A,B). As shown in Figure 2C, hsa_circ_0017620 knockdown inhibited DNA synthesis in A549 and H460 cells. Clone formation assay showed that the colony numbers of si‐hsa_circ_0017620 groups were significantly lower than that of si‐NC groups in A549 and H460 cells (Figure 2D). Moreover, MTT assay demonstrated that inhibition of hsa_circ_0017620 notably decreased cell proliferation in A549 and H460 cells (Figure 2E,F). Furthermore, si‐hsa_circ_0017620 transfection remarkably reduced Ki67 protein expression in A549 and H460 cells (Figure 2G). Thus, knockdown of hsa_circ_0017620 could inhibit cell growth in NSCLC cells. Inversely, overexpression of hsa_circ_0017620 contributed to colony formation, cell proliferation, and Ki67 protein expression in A549 and H460 cells (Figure 2 H to 2L). In a word, hsa_circ_0017620 regulated cell proliferation in NSCLC.

### 4.3 Hsa_circ_0017620 regulated cell metastasis of NSCLC

**FIGURE 3:**
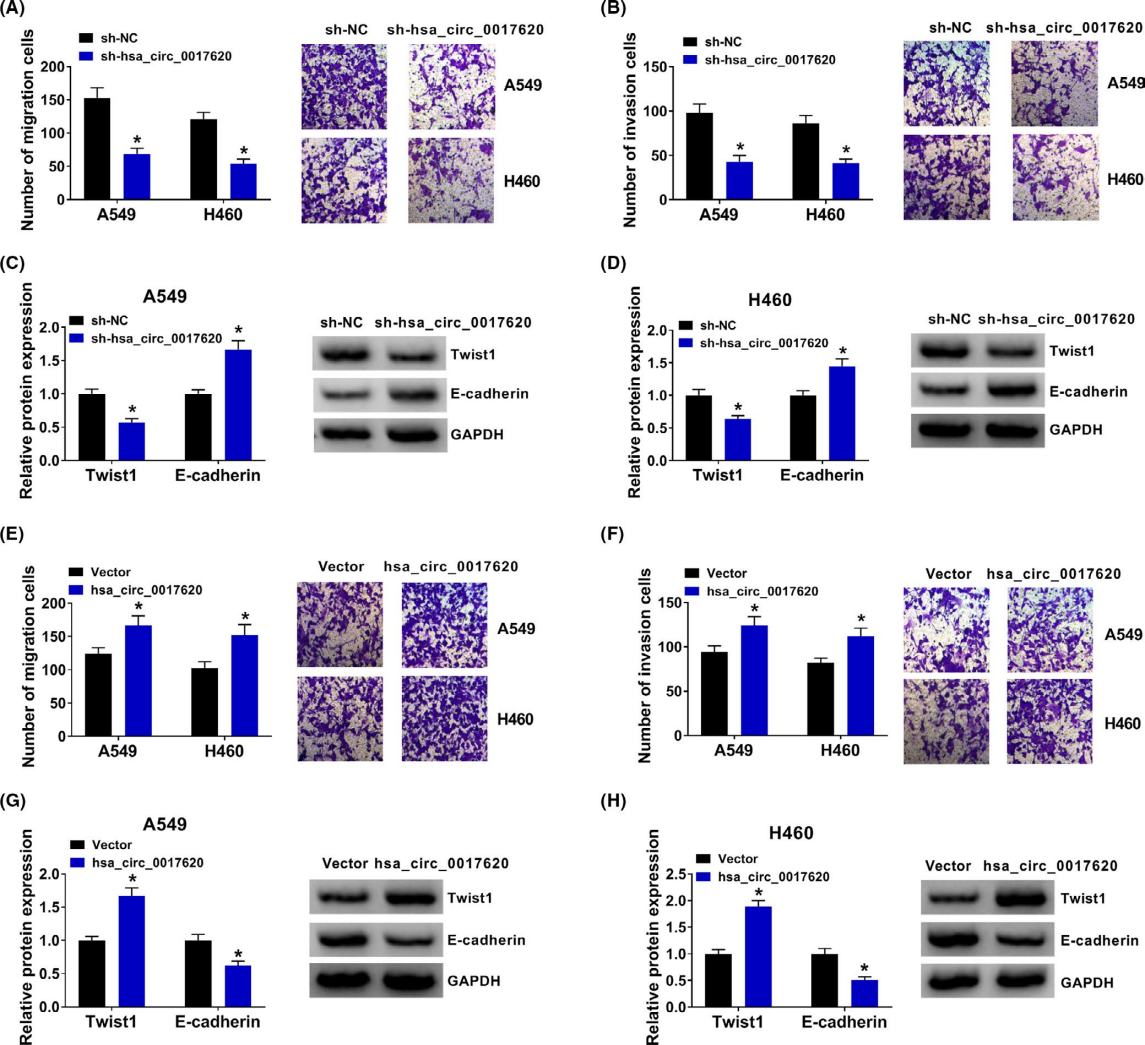
Hsa_circ_0017620 regulated cell metastasis of NSCLC. (A, B, E, F) Cell migration and invasion were measured in si‐NC, si‐hsa_circ_0017620, Vector, and hsa_circ_0017620 groups with transwell assay in A549 and H460 cells. (C, D, G, H) The protein expression of Twist1 and E‐cadherin was detected in si‐NC, si‐hsa_circ_0017620, Vector, and hsa_circ_0017620 groups with western blot in A549 and H460 cells. *p < 0.05

Next, to investigate the role of hsa_circ_0017620 on cell metastasis in NSCLC, we analyzed the capacity of cell migration and invasion in NSCLC. Transwell migration and invasion assay determined that cell migration and invasion of si‐hsa_circ_0017620 groups were significantly lower than that of si‐NC group in A549 and H460 cells (Figure 3A,B). The results of western blot showed that si‐hsa_circ_0017620 transfection inhibited Twist1 protein expression and induced E‐cadherin expression in A549 and H460 cells (Figure 3C,D). On the contrary, overexpression of hsa_circ_0017620 improved cell migration and invasion, increased Twist1 protein expression and decreased E‐cadherin protein expression in A549 and H460 cells (Figure 3E to 3H). Thus, hsa_circ_0017620 could affect cell metastasis in NSCLC.

### 4.4 Hsa_circ_0017620 directly targeted miR‐520a‐5p in NSCLC cells

**FIGURE 4:**
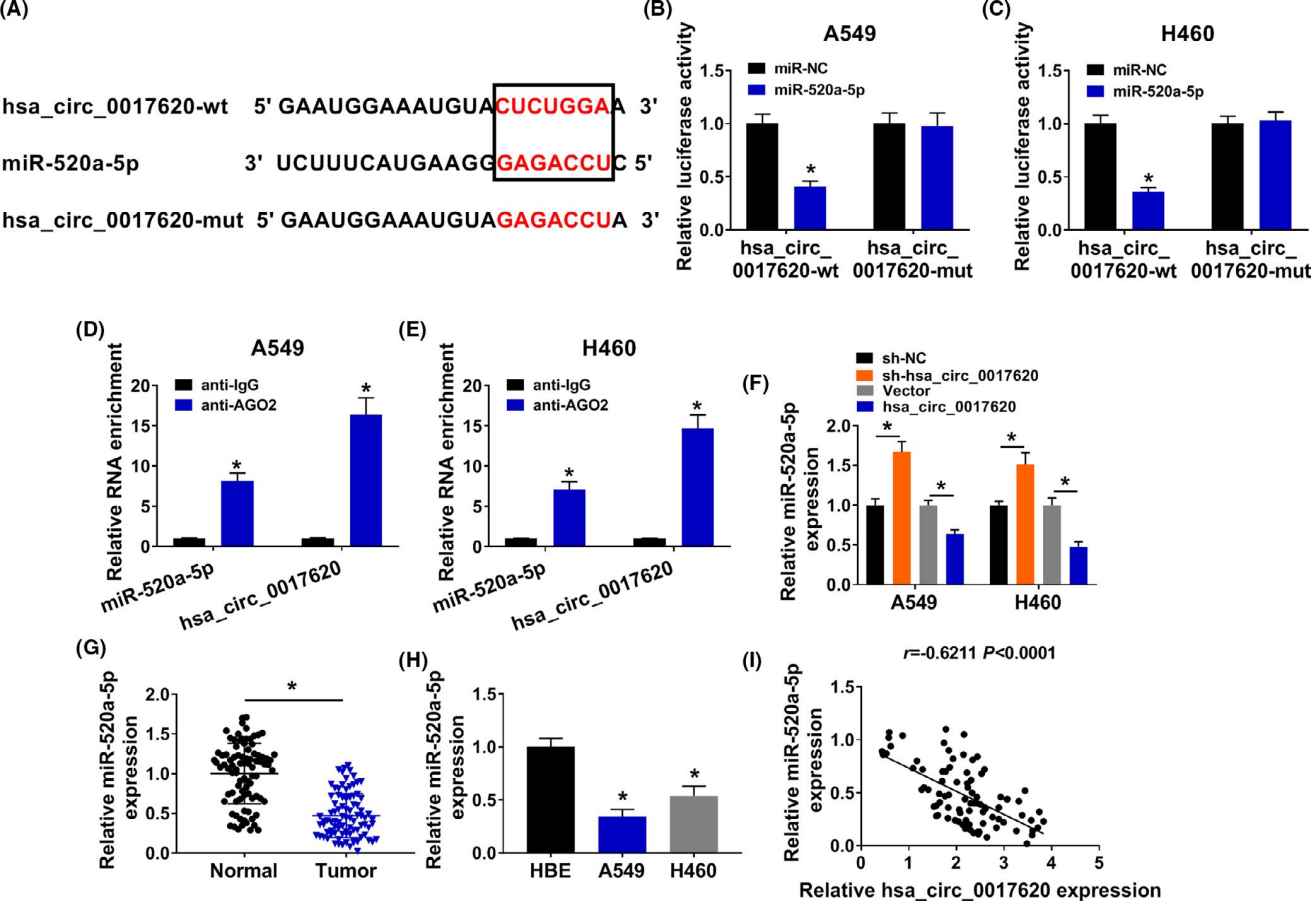
Hsa_circ_0017620 directly targeted miR‐520a‐5p in NSCLC cells. (A) The predicted binding sites of miR‐520a‐5p to the hsa_circ_0017620 sequence were shown. (B–E) Dual‐luciferase reporter and RIP assays determined that the relationship between miR‐520a‐5p and hsa_circ_0017620 in A549 and H460 cells. (F) The expression of miR‐520a‐5p was detected in si‐NC, si‐hsa_circ_0017620, Vector, and hsa_circ_0017620 groups with qRT‐PCR in A549 and H460 cells. (G) The expression of miR‐520a‐5p was detected in normal tissues and NSCLC tissues. (H) The expression of miR‐520a‐5p was detected in normal cells and NSCLC cells. (I) Pearson's correlation analysis determined the relationship between miR‐520a‐5p and hsa_circ_0017620 in NSCLC tissues. *p < 0.05

To further understand the regulatory network of hsa_circ_0017620 in NSCLC, miR‐520a‐5p was predicted to have binding sites of hsa_circ_0017620 using starBase3.0 (Figure 4A). The results of dual‐luciferase reporter assay showed that luciferase activity was reduced when hsa_circ_0017620‐wt and miR‐520a‐5p were cotransfected into A549 and H460 cells; however, the luciferase activity of hsa_circ_0017620‐mut showed no changes (Figure 4B,C). Also, we found that both miR‐520a‐5p and hsa_circ_0017620 were dramatically enriched in anti‐AGO2 group compared with their expression in anti‐IgG group (Figure 4D,E). The expression of miR‐520a‐5p was induced by si‐hsa_circ_0017620 transfection while inhibited by hsa_circ_0017620 transfection in A549 and H460 cells (Figure 4F). Moreover, compared with normal tissues and cells, miR‐520a‐5p expression was downregulated in NSCLC tissues and cells (Figure 4G,H). Pearson's correlation analysis indicated that hsa_circ_0017620 expression was negatively related to miR‐520a‐5p expression in NSCLC tissues (Figure 4I). Therefore, miR‐520a‐5p was a target miRNA of hsa_circ_0017620 in NSCLC.

### 4.5 Hsa_circ_0017620 regulated cell proliferation, migration, and invasion through targeting miR‐520a‐5p in NSCLC

**FIGURE 5:**
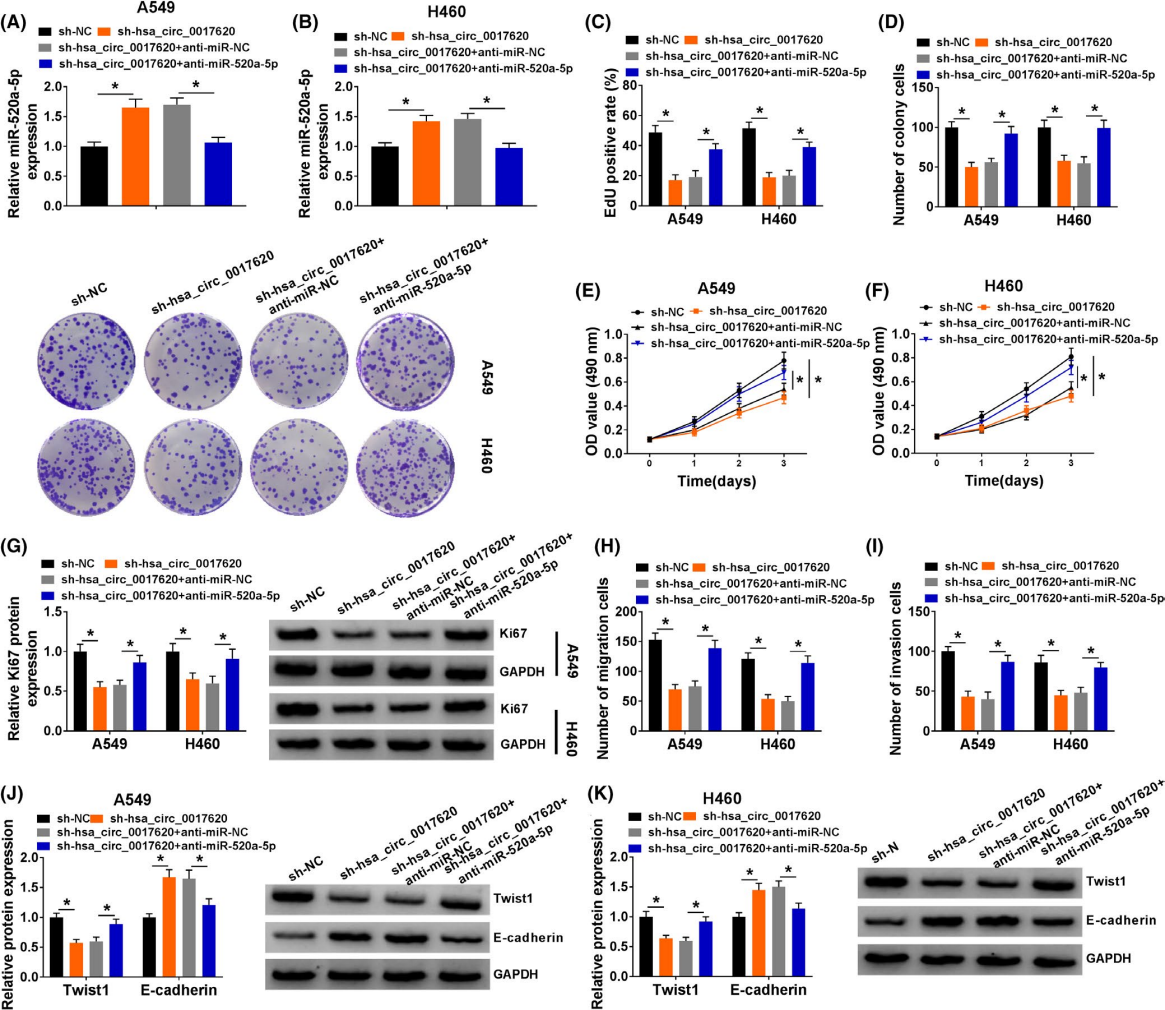
Hsa_circ_0017620 regulated cell proliferation, migration, and invasion through targeting miR‐520a‐5p in NSCLC. (A and B) The expression of miR‐520a‐5p was detected in si‐NC, si‐hsa_circ_0017620, si‐hsa_circ_0017620+anti‐miR‐NC, and si‐hsa_circ_0017620+anti‐miR‐520a‐5p groups with qRT‐PCR in A549 and H460 cells. (C) DNA synthesis was analyzed by EdU assay in A549 and H460 cells transfected with si‐NC, si‐hsa_circ_0017620, si‐hsa_circ_0017620+anti‐miR‐NC, or si‐hsa_circ_0017620+anti‐miR‐520a‐5p. (D) The number of colony cells was measured in si‐NC, si‐hsa_circ_0017620, si‐hsa_circ_0017620+anti‐miR‐NC, and si‐hsa_circ_0017620+anti‐miR‐520a‐5p groups using colony formation assay in A549 and H460 cells. (E and F) Cell proliferation was measured using MTT assay in si‐NC, si‐hsa_circ_0017620, si‐hsa_circ_0017620+anti‐miR‐NC, and si‐hsa_circ_0017620+anti‐miR‐520a‐5p groups in A549 and H460 cells. (G) The protein expression of Ki67 was detected with western blot in si‐NC, si‐hsa_circ_0017620, si‐hsa_circ_0017620+anti‐miR‐NC, and si‐hsa_circ_0017620+anti‐miR‐520a‐5p groups in A549 and H460 cells. (H and I) Cell migration and invasion were measured with transwell assay in si‐NC, si‐hsa_circ_0017620, si‐hsa_circ_0017620+anti‐miR‐NC, and si‐hsa_circ_0017620+anti‐miR‐520a‐5p groups in A549 and H460 cells. (J and K) The protein expression of Twist1 and E‐cadherin was detected with western blot in si‐NC, si‐hsa_circ_0017620, si‐hsa_circ_0017620+anti‐miR‐NC, and si‐hsa_circ_0017620+anti‐miR‐520a‐5p groups in A549 and H460 cells. *p < 0.05

To further verify the role of hsa_circ_0017620 and miR‐520a‐5p in NSCLC, we transfected si‐NC, si‐crc_SFMBT2, si‐hsa_circ_0017620+anti‐miR‐NC, and si‐hsa_circ_0017620+anti‐miR‐520a‐5p into A549 and H460 cells. The results showed that knockdown of hsa_circ_0017620 significantly induced miR‐520a‐5p expression, which was impaired by inhibiting miR‐520a‐5p in A549 and H460 cells (Figure 5A,B). Cell proliferation was notably suppressed by si‐hsa_circ_0017620 transfection, while this effect was reversed by downregulating miR‐520a‐5p expression in A549 and H460 cells (Figure 5C–F). Moreover, western blot assay showed that anti‐miR‐520a‐5p transfection could rescue the inhibitory effects of low hsa_circ_0017620 expression on Ki67 protein expression in A549 and H460 cells (Figure 5G). Transwell assay determined that cell migration and invasion were remarkably reduced by inhibition of hsa_circ_0017620, which were reversed by down expression of miR‐520a‐5p in A549 and H460 cells (Figure 5H,I). Twist1 and E‐cadherin were markers of EMT. As shown in Figure 5J,K, knockdown of hsa_circ_0017620 inhibited Twist protein expression while promoted E‐cadherin protein expression, which was reversed by suppression of miR‐520a‐5p in A549 and H460 cells. Thus, hsa_circ_0017620 affected cell growth by targeting miR‐520a‐5p in NSCLC.

### 4.6 KRT5 was a target mRNA of miR‐520a‐5p

**FIGURE 6:**
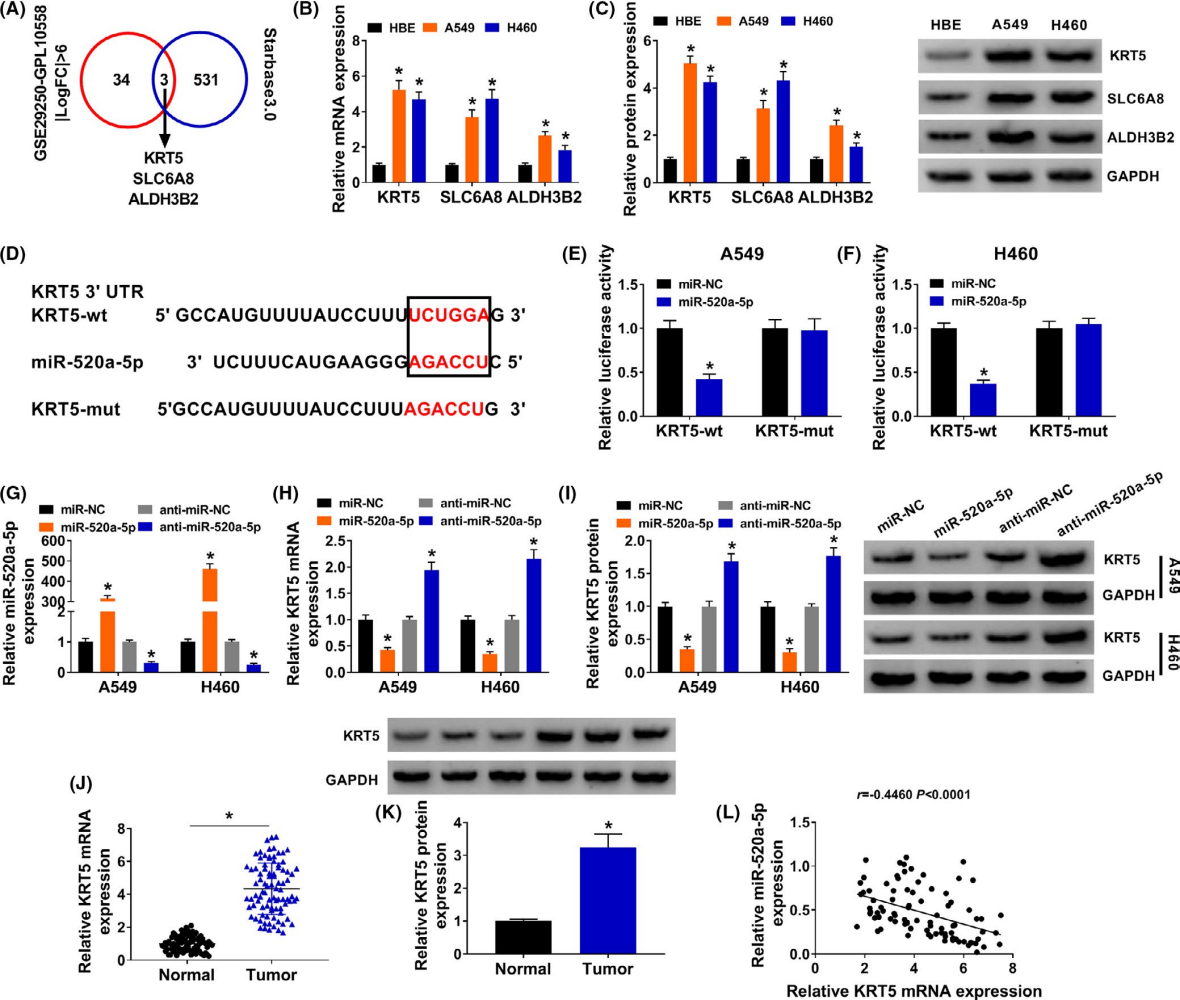
KRT5 was a target mRNA of miR‐520a‐5p. (A) Venn diagram between GSE29250‐GPL10558 and Starbase3.0 dataset (| logFC |> 6). (B and C) The results of qRT‐PCR and western blot showed that the mRNA and protein expressions of KRT5, SLC6A8, and ALDH3B2 were significantly reduced in NSCLC tissues. (D) The predicted binding sites of miR‐520a‐5p to the KRT5 sequence were shown. (E and F) Dual‐luciferase reporter assay determined that the relationship between miR‐520a‐5p and KRT5 in A549 and H460 cells. (G) The expression of miR‐520a‐5p was detected in miR‐NC, miR‐520a‐5p, anti‐miR‐NC, and anti‐miR‐520a‐5p groups with qRT‐PCR in A549 and H460 cells. (H and I) The mRNA and protein expression of KRT5 were detected in miR‐NC, miR‐520a‐5p, anti‐miR‐NC, and anti‐miR‐520a‐5p groups with qRT‐PCR and western blot in A549 and H460 cells. (J and K) The mRNA and protein expression of KRT5 were detected in normal and tumor in NSCLC with qRT‐PCR and western blot. (L) Pearson's correlation analysis determined the relationship between miR‐520a‐5p and KRT5 in NSCLC tissues. *p < 0.05

As shown in Figure 6A, the intersection of GSE29250‐GPL10558 (|LogFC| >6) and Starbase3.0 dataset were KRT5, SLC6A8, and ALDH3B2, which might be the target mRNAs of miR‐520a‐5p in NSCLC. The results of qRT‐PCR and western blot showed that the mRNA and protein expressions of KRT5, SLC6A8, and ALDH3B2 were significantly induced in NSCLC cells, among which the selected average expression of KRT5 was the largest (Figure 6B,C). We also found that KRT5 had binding sites of miR‐520a‐5p (Figure 6D). Dual‐luciferase reporter assay showed that when the miR‐520a‐5p bound to KRT5‐wt, luciferase activity was significantly decreased in A549 and H460 cells (Figure 6E,F). As shown in Figure 6G, miR‐520a‐5p transfection induced miR‐520a‐5p expression, while anti‐miR‐520a‐5p transfection significantly inhibited miR‐520a‐5p expression in A549 and H460 cells. qRT‐PCR and western blot determined that miR‐520a‐5p transfection inhibited KRT5 mRNA and protein expression, while anti‐miR‐520a‐5p transfection significantly increased KRT5 mRNA and protein expression in A549 and H460 cells (Figure 6H,I). Besides, the mRNA and protein expression of KRT5 were induced in tumor tissues compared with normal tissues (Figure 6J,K). Pearson's correlation analysis showed that miR‐520a‐5p expression was negatively related to KRT5 expression in NSCLC tissues (Figure 6L). Thus, miR‐520a‐5p directly targeted KRT5 in NSCLC.

### 4.7 Hsa_circ_0017620 regulated cell progression through modulating KRT5 in NSCLC cells

**FIGURE 7:**
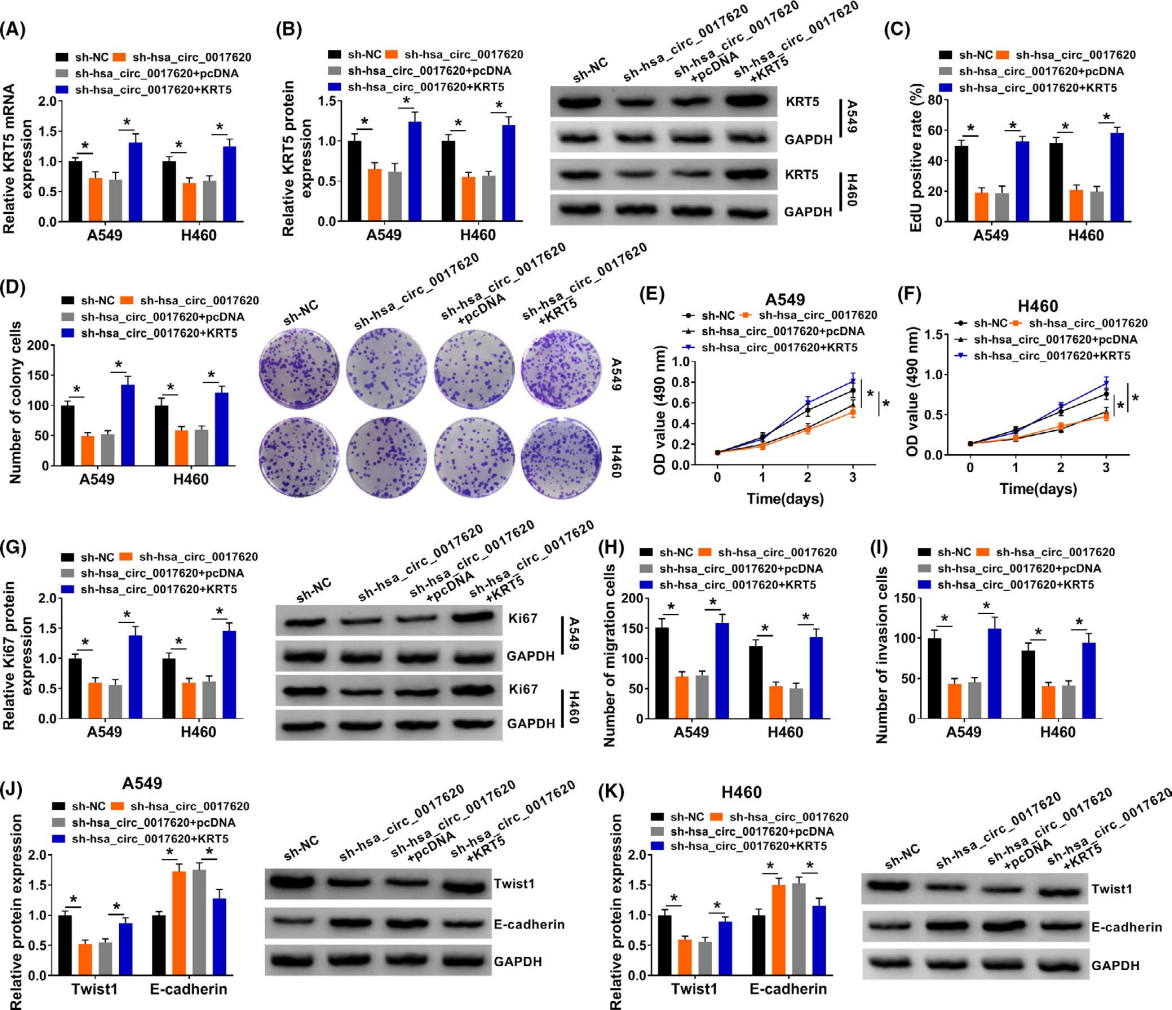
Hsa_circ_0017620 regulated cell progression through modulating KRT5 in NSCLC cells. (A and B) The mRNA and protein expression of KRT5 were detected in si‐NC, si‐hsa_circ_0017620, si‐hsa_circ_0017620+pcDNA, and si‐hsa_circ_0017620+KRT5 groups with qRT‐PCR and western blot in A549 and H460 cells. (C) DNA synthesis was analyzed by EdU assay in A549 and H460 cells transfected with si‐NC, si‐hsa_circ_0017620, si‐hsa_circ_0017620+pcDNA, or si‐hsa_circ_0017620+KRT5. (D) The number of colony cells was measured using colony formation assay in si‐NC, si‐hsa_circ_0017620, si‐hsa_circ_0017620+pcDNA, and si‐hsa_circ_0017620+KRT5 groups. (E and F) Cell proliferation was measured in si‐NC, si‐hsa_circ_0017620, si‐hsa_circ_0017620+pcDNA, and si‐hsa_circ_0017620+KRT5 groups with MTT assay in A549 and H460 cells. (G) The protein expression of Ki67 was measured with western blot in si‐NC, si‐hsa_circ_0017620, si‐hsa_circ_0017620+pcDNA, and si‐hsa_circ_0017620+KRT5 groups in A549 and H460 cells. (H and I) Cell migration and invasion were assessed with transwell assay in si‐NC, si‐hsa_circ_0017620, si‐hsa_circ_0017620+pcDNA, and si‐hsa_circ_0017620+KRT5 groups in A549 and H460 cells. (J and K) The protein expression of Twist1 and E‐cadherin was detected with western blot in si‐NC, si‐hsa_circ_0017620, si‐hsa_circ_0017620+pcDNA, and si‐hsa_circ_0017620+KRT5 groups in A549 and H460 cells. *p < 0.05

To further explore the function of hsa_circ_0017620 and KRT5 in NSCLC, we transfected si‐NC, si‐hsa_circ_0017620, si‐hsa_circ_0017620+pcDNA, and si‐hsa_circ_0017620+KRT5 into A549 and H460 cells and the results showed that compared with si‐NC groups, KRT5 mRNA and protein expression were significantly inhibited in si‐hsa_circ_0017620 groups in A549 cells and H460 cells (Figure 7A,B). However, KRT5 mRNA and protein expression were higher in si‐hsa_circ_0017620+KRT5 group than that in si‐crc_SFMBT2+pcDNA groups (Figure 7A,B). In addition, si‐hsa_circ_0017620 transfection inhibited cell progression, which was reversed by overexpression of KRT5 in A549 and H460 cells (Figure 7C‐F). As shown in Figure 7H,I, inhibition of hsa_circ_0017620 notably reduced the capacities of cell invasion and migration, which was impaired by the promotion of KRT5 in A549 and H460 cells. Western blot determined that si‐hsa_circ_0017620 transfection inhibited Ki67 and Twist protein expression, while promoted E‐cadherin protein expression, which was rescued by upregulating KRT5 expression in A549 and H460 cells (Figure 7G, J, and K). Therefore, overexpression of KRT5 could reverse the inhibitory effects of low hsa_circ_0017620 expression on cell growth in NSCLC cells.

### 4.8 Hsa_circ_0017620 silencing inhibited tumor growth in vivo

**FIGURE 8:**
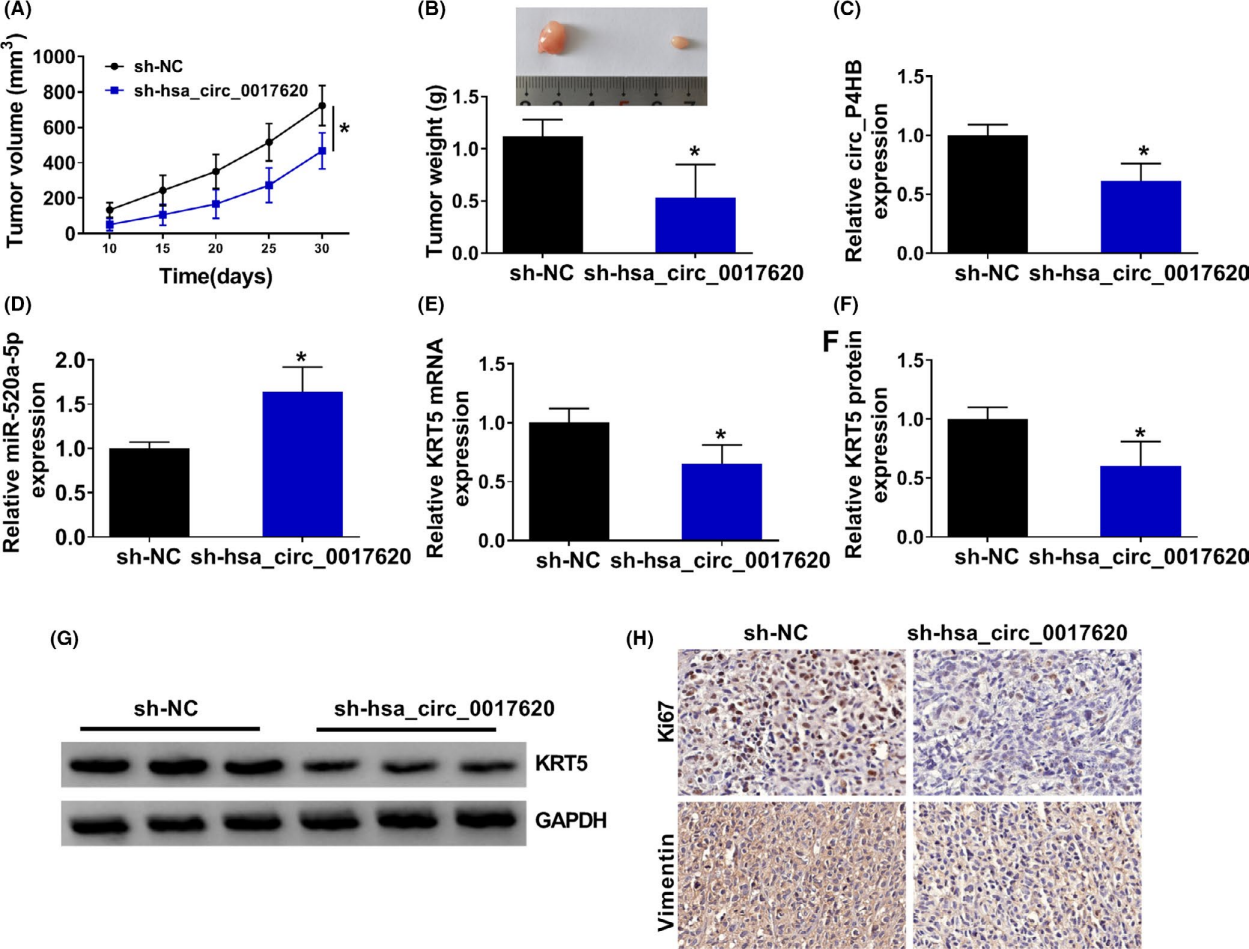
Hsa_circ_0017620 silencing inhibited tumor growth *in vivo*. (A) Growth curves of tumor volume in sh‐NC and sh‐hsa_circ_0017620 groups. (B) The tumor weight was measured in sh‐NC and sh‐hsa_circ_0017620 groups. (C and D) The expression of hsa_circ_0017620 and miR‐520a‐5p was measured in sh‐NC and sh‐hsa_circ_0017620 groups with qRT‐PCR. (E–G) The mRNA and protein expression of KRT5 were measured in sh‐NC and sh‐hsa_circ_0017620 groups with western blot. (H) The positive expression rates of Ki67 and Vimentin in the forming tumors from sh‐NC and sh‐hsa_circ_0017620 groups were detected by IHC assay. *p < 0.05

To prove the function of hsa_circ_0017620 *in vivo*, we constructed and obtained the stably transfected cell lines (sh‐NC and sh‐hsa_circ_0017620) and injected sh‐NC and sh‐hsa_circ_0017620 subcutaneously into mice. Mice tumor size was measured every 5 days until 30 days after transfection. The mice were then killed and the tumors were weighed in the mice. The results showed that inhibition of hsa_circ_0017620 effectively inhibited tumor growth (Figure 8A,B). Furthermore, we found that sh‐hsa_circ_0017620 transfection inhibited the expression of hsa_circ_0017620 and the mRNA and protein expression of KRT5 while promoted miR‐520a‐5p expression *in vivo* (Figure 8C‐G). Meanwhile, IHC assay showed that hsa_circ_0017620 depletion inhibited the positive expression rates of Ki67 and Vimentin (Figure 8H). Thus, down expression of hsa_circ_0017620 could inhibit tumor growth of NSCLC *in vivo*.

## 5 DISCUSSION

Accumulating evidence indicated that circRNA is a very important regulator in the development of tumor cells, which sponges miRNA to regulate mRNA to affect the metabolism and development of tumor cells.^14^, ^17^ Cell proliferation, migration, and invasion are important characteristics of tumor cell development and are very important in the study of tumor characteristics. Various evidence suggests that circRNA is significantly related to the progression, diagnosis, and prognosis of NSCLC.^18^, ^19^, ^20^ For example, hsa_circ_0067934 silencing suppressed cell proliferation, migration, and invasion.^21^ Moreover, hsa_circ_0067934 and hsa_circ_0007534 had been verified to be closely correlated with an unfavorable prognosis.^20^, ^21^ Previous evidence has suggested that hsa_circ_0017620 promotes gastric cancer cell proliferation and metastasis through miR‐224‐5p.^22^ Recent data indicated the promoting effect of the circRNA on the malignant progression of esophageal cancer.^23^ Herein, hsa_circ_0017620 was highly expressed in NSCLC and significantly promoted proliferation and mobility of NSCLC cells.

It is generally known that circRNAs were involved in tumor development by binding to miRNAs to regulate mRNAs.^14^ In NSCLC, hsa_circ_0000003 enhanced cell proliferation and metastasis via miR‐338‐3p/IRS2.^24^ In this study, hsa_circ_0017620 bound to miR‐520a‐5p to affect KRT5 expression in NSCLC. MiR‐520a‐5p was lowly expressed in NSCLC specimens, while KRT5 was highly expressed. Studies have shown that miR‐520a‐5p is lowly expressed in breast cancer and NSCLC and participates in regulation of cell proliferation, migration, invasion, and apoptosis.^25^, ^26^ Keratin 5 (KRT5), located at chromosome 12, also had been certified to be involved in cancer progression.^27^ For example, KRT5 in serous ovarian cancer was associated with chemotherapy resistance and cancer recurrence.^28^ Therefore, we predicted that hsa_circ_0017620/miR‐520a‐5p/KRT5 axis was involved in NCSLC formation and cellular progression.

In previous studies, the regulatory pathways of circRNAs participated in tumor metabolism and growth mechanisms.^29^, ^30^ For example, hsa_circ_0020397 affected cell viability, apoptosis, and invasion through sponging miR‐138 to modulate TERT and PD‐L in colorectal cancer.^31^ Hsa_circ_0046264 enhanced BRCA2 expression to inhibit lung cancer via targeting miR‐1245.^32^ In this study, we used a reverse experiment to demonstrate the potential regulatory mechanism of hsa_circ_0017620 in NSCLC. Low expression of miR‐520a‐5p or high expression of KRT5 can significantly reverse si‐hsa_circ_0017620‐reduced proliferation and mobility of NSCLC cells. Moreover, we also demonstrated in mice that reducing hsa_circ_0017620 expression significantly inhibited tumor tumorigenesis. Hsa_circ_0017620/miR‐520a‐5p/KRT5 axis was a new and important path that affected the development and development of NSCLC.

## 6 CONCLUSION

However, one circRNA or miRNA targeted multiple miRNAs or mRNAs, and multiple circRNAs or miRNAs targeted one miRNA and mRNA in cancers, leading to indeed complexity of research on the regulatory mechanism of circRNA.^33^, ^34^, ^35^, ^36^, ^37^ Therefore, we needed to find more regulatory pathways for hsa_circ_0017620 in NSCLC to improve our understanding about the pathogenesis of NSCLC.

## CONFLICT OF INTEREST

The authors declare that they have no financial conflicts of interest.

## Data Availability

Not Applicable.
